# Time-Dependent Regional Myocardial Strains in Patients with Heart Failure with a Preserved Ejection Fraction

**DOI:** 10.1155/2016/8957307

**Published:** 2016-03-03

**Authors:** Shane P. Smith, Timothy W. Secomb, Brian D. Hong, Michael J. Moulton

**Affiliations:** ^1^Division of Cardiothoracic Surgery, Department of Surgery, University of Nebraska Medical Center, Omaha, NE 68198, USA; ^2^Department of Physiology, University of Arizona, Tucson, AZ 85724, USA; ^3^Program in Applied Mathematics, University of Arizona, Tucson, AZ 85721, USA

## Abstract

*Objectives*. To better understand the etiology of HFpEF in a controlled human population, regional time-varying strains were computed using echocardiography speckle tracking in patients with heart failure with a preserved ejection fraction and normal subjects.* Methods*. Eleven normal volunteers and ten patients with echo-graded diastolic dysfunction and symptoms of heart failure were imaged with echocardiography and longitudinal, circumferential, and rotational strains were determined using speckle-tracking. Diastolic strain rate was also determined. Patient demographics and echo-derived flows, volumes, and pressures were recorded.* Results*. Peak longitudinal and circumferential strain was globally reduced in patients (*p* < 0.001), when compared to controls. The patients attained peak longitudinal and circumferential strain at a consistently later point in systole than controls. Rotational strains were not different in most LV regions. Early diastolic strain rate was significantly reduced in the patients (*p* < 0.001). LV mass and wall thickness were significantly increased in the patients; however ejection fraction was preserved and stroke volume was diminished (*p* < 0.001).* Conclusions*. This study shows that patients with HFpEF have reduced early diastolic strain rate and reduced peak strain that is regionally homogeneous and that they also utilize a longer fraction of systole to achieve peak axial strains.

## 1. Introduction

Heart failure with a preserved ejection fraction (HFpEF) now accounts for half of patients seen by physicians who have clinical heart failure symptoms [1]. Epidemiologic data suggests that these patients have life expectancy and hospital admission rates similar to patients with reduced systolic function (HFrEF) [1]. Several studies show that the end organ and whole body phenotype of the disease are similar to HFrEF: increased salt and water retention with increased venous blood volume, neurohormonal activation, pulmonary congestion with increased filling pressures and elevated brain natriuretic peptide (BNP) [2]. Increasingly detailed studies in animal models and humans have developed sufficient data to propose a hypothesis for the disease. These detailed studies of pressure-volume relationships, myocardial wall stress, and diastolic and systolic strain rate suggest that HFpEF is a disease of increased diastolic stiffness, possibly due to a combination of delayed relaxation and intrinsic stiffness of the myocardium [3]. More recent studies in animal models attempted to explore the cellular mechanism of increased stiffness. These studies looked at the two most likely sources of increased stiffness in the myocardium: altered Titin N2B “spring” isoforms leading to increased intrinsic myocyte stiffness and changes in the extracellular collagen matrix [4]. Intrinsic myocyte stiffness related to Titin isoform changes resulted in phenotypic changes consistent with HFpEF in the absence of any changes in collagen matrix [4, 5].

There is great difficulty in translating these detailed experimental findings into an understanding of HFpEF in humans. Several randomized controlled clinical trials [6–8] examining proposed treatments (angiotensin antagonists, beta blocker, and digoxin) for HFpEF were negative and to date there is no effective treatment that alters life expectancy or hospitalization rates for patients with HFpEF. Symptomatic relief with diuretics is the only effective therapy. Some of the difficulty in treating the disease occurs because HFpEF may have diverse causes in humans compared with animal models. There are associations of HFpEF to hypertension, diabetes, obesity, aging, and coronary artery disease [1]. It is doubtful that there is a single underlying mechanism for the disease in humans. Since measurements of muscle properties and molecular assessments of Titin, for example, are not feasible in large human populations, a technique like echocardiography speckle tracking is particularly useful for making noninvasive, detailed regional measurements of heart motion. Older techniques like pressure-volume measurements provided good evidence of increased stiffness of the entire LV but offered no possibility of further elucidation of a mechanism and are invasive [2]. Previous studies with echocardiography speckle tracking in HFpEF patients only utilized one number—peak strain or peak strain rate—and did not directly examine the time-varying pattern of strain available throughout the cardiac cycle and these studies did not quantify the variability in strain data throughout the cycle [9, 10]. The temporal resolution of echo is one of its great assets when compared with competing imaging methods like MRI and therefore it is likely that changes in the time-varying nature of strains might be more reliable and informative than regional changes in strain or the magnitude of strains.

Echocardiography is readily available to even the most ill patients with heart failure and can be accomplished in reasonable times at the location where the patients are treated. What is not known is whether these detailed measurements of heart motion are sufficiently accurate and sensitive to the changes in the heart muscle that occur with HFpEF to shed light on regional or even molecular mechanisms. In this paper we analyze the results of regional speckle tracking measurements of longitudinal, circumferential, and rotational strains in patients with HFpEF and in a control population. The magnitude of the error seen in the speckle tracking echo strains is quantified. The time-varying pattern of strains for the entire cardiac cycle is then examined to gain information about the mechanism underlying LV dysfunction in HFpEF. The longer term goal of this work is to determine whether progressively detailed and validated measurements of LV wall strains in a larger subset of patients with HFpEF can provide evidence supporting hypothesized molecular mechanisms of HFpEF. For example, it may be possible to distinguish between increased Titin N2B terminal spring stiffness and increased collagen stiffness through distinctive resulting patterns of LV motion.

## 2. Methods

### 2.1. Human Subjects

Ten patients with diagnosed HFpEF were enrolled in the study. Each patient had stable heart failure symptoms and was in New York Heart Association (NYHA) Classes II-III. We enrolled patients who met the criteria for HFpEF published by Vasan and Levy and [11] who were known to have Grade II or greater diastolic dysfunction as reflected by their Doppler mitral inflow pattern. Patients with acute or decompensated heart failure were not included. Patients who had greater than trace valvular regurgitation or who had mild or greater stenosis of a heart valve were excluded. To further obtain a more homogenous patient population, those patients with atrial fibrillation were excluded. These patients were compared with eleven control patients who were employees of the University of Nebraska Medical Center and volunteered for enrollment. These patients had no symptoms of cardiac disease or failure and all had normal ejection fraction. Myocardial dysfunction, valvular disease, and atrial fibrillation were all ruled out in the control population with echocardiography (ECHO) and electrocardiogram (ECG).

The Institutional Review Board of the University of Nebraska Medical Center approved the study protocol. All procedures performed were in accordance with the committee on human experimentation (institutional and national) and with the Helsinki Declaration of 1975, as revised in 2000. Written informed consent was obtained from the volunteers and patients before their addition to the study.

### 2.2. Echocardiography Image Acquisition

A standard echocardiography protocol was utilized. Apical, mid, and basal short axis (SA) regions were scanned using a high frame rate (70–80 frames/s) second harmonic B-mode transducer (transmit/receive 1.4/2.8 MHz) with a Phillips S5-1 ultrasound probe with Hpen on a IE33 echocardiography machine (Phillips Medical Systems, Amsterdam, Netherlands).

**Figure 1:**
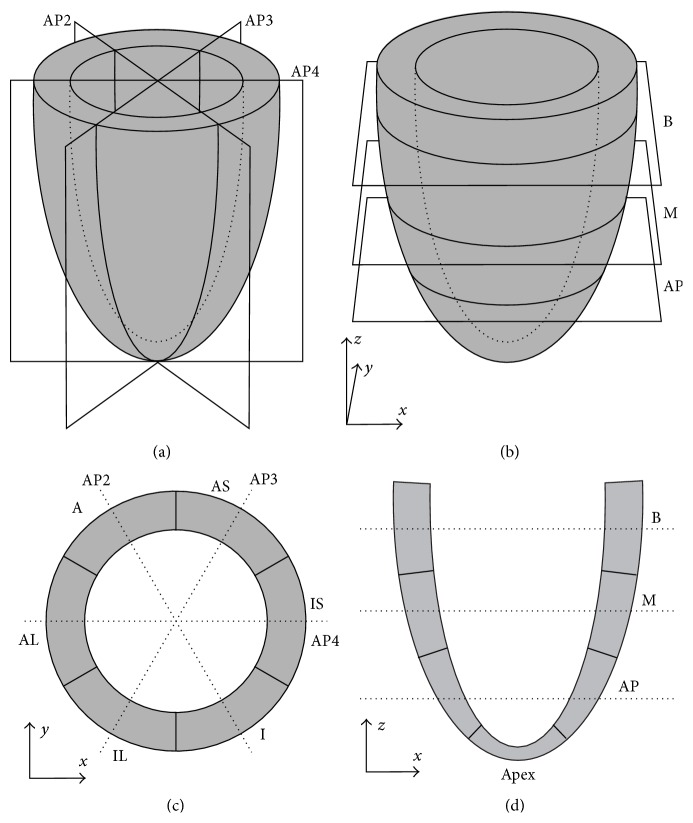
Definition of the imaging planes and regions used in the echocardiographic imaging of the LV. (a) Long axis imaging planes. AP2: apical two-chamber view; AP3: apical three-chamber view; AP4: apical four-chamber view. (b) Short axis imaging planes. B: basal; M: mid, AP: apical. (c) Short axis imagining regions, as viewed from the base of the heart. The regions appear in opposite order to those in the apical view used in imaging. AS: anterior septal; A: anterior; AL: anterior lateral; IL: inferior lateral; I: inferior; IS: inferior septal. (d) Long axis imaging regions.

The long axis (LA) images were standard four-chamber (AP4), three-chamber (AP3), and two-chamber (AP2) views of the LV. These images represent sections in planes orientated at approximately 60° angles from each other. The following definitions were assigned to the SA imaging planes: at the basal level, the mitral valve is in view. The midlevel image was obtained at the upper portion of the papillary muscles. At the apical level, the LV cavity alone is visualized with no papillary muscles. The imaging planes and regions are shown schematically in Figure 1.

ECG gating was employed for each image view and two heartbeats were obtained. Customized software (Phillips QLab 8.4) on a personal computer workstation was used for subsequent off-line analysis of ventricular geometry and strains obtained by speckle tracking after image acquisition.

### 2.3. Image Analysis: Time-Dependent Myocardial Strains

The SA and LA images were analyzed using software designed to compute cardiac motion and strain components (Phillips QLab 8.4). Endocardial and epicardial borders were manually selected on a reference image of each SA and LA view. Borders were selected on a still frame of the heart in end systole. Each SA image was subdivided into six circumferential regions: anterior (A), anterior septal (AS), inferior septal (IS), inferior (I), inferior lateral (IL), and anterior lateral (AL) (Figure 1(c)). Each LA axis image was subdivided into basal (B), mid (M), and apical (AP) regions (Figure 1(d)). These were in turn divided into endocardial and epicardial regions, yielding a total of 36 regions for strain analysis based on both SA and LA views. These regions were tracked automatically by the software at the echo frame rate, giving approximately 70–80 time points for each heartbeat. Each region was subdivided into triangular regions of interest (ROI). Maximal image acquisition was achieved by using the finest triangular mesh available in the QLab program, producing the maximum amount of ROI. In each ROI, the speckle tracking algorithm attempted to track natural acoustic markers from frame to frame. Spatial and temporal smoothing was employed and a set of tissue displacements was computed for each triangular element at each time point. Engineering strains were computed from these displacements. Average circumferential and longitudinal strains were obtained for each of the 36 regions from SA and LA images, respectively. Regional rotations (in degrees) were also obtained from SA images.

### 2.4. Data Analysis: Base, Mid, and Apical Differences

For longitudinal strain, the B, M, and AP regions were obtained for each patient. For circumferential strain, the six regions of A, AS, IS, I, IL, and AL were combined into 3 groups based on their level of acquisition: basal, mid, and apical. The same was done for regional rotation.

Each region produced strain or rotation over two heartbeats. The data were then normalized to a single cardiac cycle, eliminating the variability in time with multiple patients. The strain data were then subdivided into twenty-five fractions of one cardiac cycle and each patient's average for strain within that fraction contributed to an overall average for strain at that point for the study group. Eleven subjects for the control group and ten subjects for the diastolic dysfunction group contributed to each of the twenty-five fractions of a single cardiac cycle.

### 2.5. Doppler Flow Analysis

In addition to image acquisition, Doppler flow analysis was also performed to assess any subsequent myocardial and/or valvular disease states in the control and diastolic dysfunction populations. For each patient, cuff blood pressure was taken before transthoracic echocardiography. Heart rate was then measured with ECG. Doppler flow was recorded continuously in the mitral and aortic valves using mitral valve (MV) closing as a point of reference. The following values were then recorded: MV closure time, isovolumetric relaxation time (IVRT), aortic valve (AR) opening time, AV close time, MV open time, and R-R interval. The TR jet was measured to assess tricuspid regurgitation. IVC pressure was recorded with six heartbeats on IVC with a sniff. The transducer was placed in a subcostal position. Then using M mode, the maximal and minimal diameter of the IVC are recorded. There is a known correlation between IVC diameter change and CVP [12]. Left atrial pressure was obtained with the equation left atrial pressure = 1.2(*E*/*E*′) + 2, where *E*/*E*′ is the ratio of the early (*E*) ventricular filling velocity to (*E*′) the longitudinal displacement of the mitral annulus [13]. Cardiac index was obtained using body surface area (BSA), left ventricular outflow tract diameter (LVOT dia), and left ventricular outflow tract velocity time integral (LVOT VTI).

### 2.6. Geometric Data

Utilizing the fitted ellipsoidal models described previously [14], LV models were constructed that best fit the observed geometry of the LV at ED and ES. Stroke volume (SV), LV mass, diastolic wall thickness, and ejection fraction (EF) were computed for the controls and patients with HFpEF.

### 2.7. Early Diastolic Strain Rate

**Figure 2:**
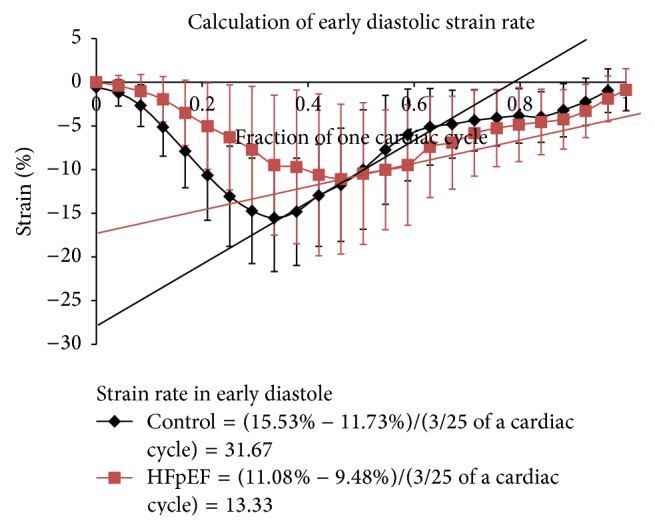
Graphical description of the method for computing early diastolic strain rate (SRed).

To assess the rate of early diastolic strains, the slope of the strain curves starting at the peak point of systole was obtained. The abscissa of the slope was defined as the first 3/25th of the cardiac cycle after the peak strain value. Then early diastolic strain rate (SRed) for the longitudinal strain was defined as [longitudinal strain (peak strain time + 3/25) − longitudinal strain (peak strain time)]/(3/25) and is computed similarly to circumferential strain (Figure 2). The endocardial and epicardial base, mid, and apex regions were then averaged together and the control population was compared to the HFpEF patients.

### 2.8. Time to Peak Systolic Strain

To test whether HFpEF patients demonstrate delayed time to peak systolic strain, the time to peak systolic strain was computed as the percentage of the cardiac cycle to reach peak systolic strain from end diastole. Because HFpEF and controls had different heart rates we used data from Weisdorf and Spodick [15] and Mertens et al. [16] to normalize the control data to be the same HR as HFpEF patients.

### 2.9. Statistical Analysis

Two sample comparisons of the control and HFpEF strain data were performed using *t*-test if the variables were normally distributed and the Mann-Whitney *U* test if not normally distributed. Chi square was employed for categorical data. The same procedure was employed to test hemodynamic and geometric data between groups. A *p* value less than 0.05 was considered statistically significant. The data were fully accessible to the authors of this paper, who take full responsibility for the integrity of the data.

## 3. Results

### 3.1. Patient Characteristics

The study included 14 women and 7 men with a median age of 50 years and a range of 23–71 years. There were more women in the control group, while the patients with HFpEF were evenly split. The HFpEF patients were older than the controls and they also had a higher frequency of hypertension, diabetes, and hyperlipidemia.  Table 1 displays risk factors, demographics, and comorbidities of controls versus those with HFpEF.

### 3.2. Echocardiography Images

**Figure 3:**
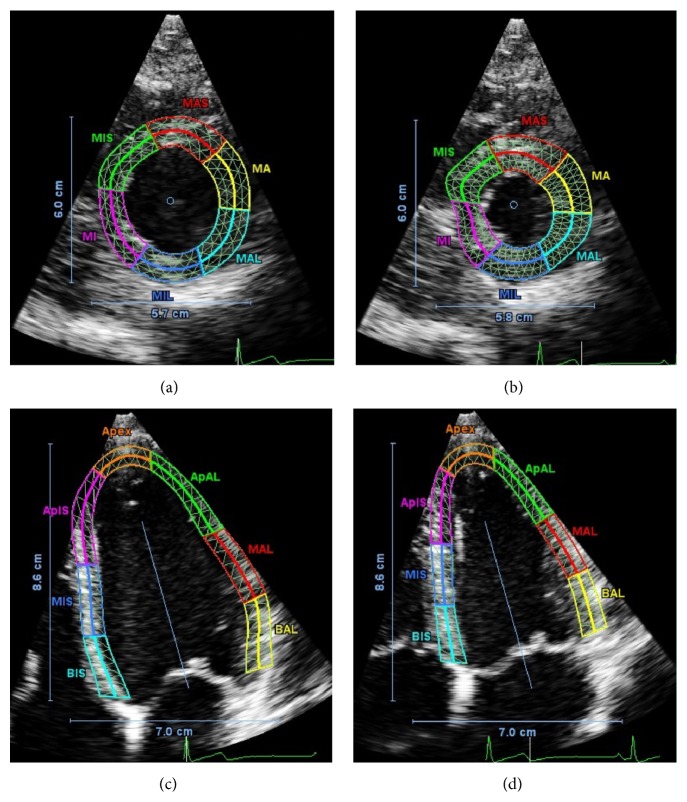
Example images (short axis (a, b) and long axis (c, d)) from echocardiography of a single control patient. SA images are at the midlevel plane, as viewed from the apex of the heart. LA images are in the four-chamber plane (AP4). The colored overlays indicate the myocardial regions considered in the estimation of regional LV strain and rotation as defined in Figure 1. The labels on each region indicate the subdivision in both longitudinal and circumferential directions. Preceding a region, “A” represents apex, “M” represents mid, and “B” represents base. For example, “MAL” represents mid plane, anterior lateral region. Each region is further subdivided into the epicardial and endocardial regions by the bold solid line in the center of each colored region.

Examples of echocardiography images from a single control patient are displayed in Figure 3. Short axis images (Figures 3(a) and 3(b)) and long axis images (Figures 3(c) and 3(d)) are displayed. The color overlay shows the myocardial boundaries as manually identified at end diastole (Figures 3(a) and 3(c)) and the boundary tracked by the QLab imaging software at end systole (Figures 3(b) and 3(d)). The overlay also shows the mesh of triangular regions of interest (ROI) that are used in the speckle tracking algorithm.

### 3.3. Cardiac Performance by Echocardiography

A full echocardiographic examination was performed for each of the patients (Table 2).

### 3.4. Geometric Data

Utilizing the fitted ellipsoidal ED and ES patient-specific models defined in [14], LV mass was 126 ± 31 g in controls versus 142 ± 36 g in HFpEF and SV was 49 ± 11 cc versus 31 ± 14 cc; EF was 56 ± 6% versus 50 ± 6% and end diastolic wall thickness was 0.98 ± 0.1 cm versus 1.18 ± 0.13 cm in controls versus HFpEF (Table 2). LV mass and ED wall thickness are significantly increased in HFpEF (*p* < 0.01) while EF is preserved and stroke volume is reduced (*p* < 0.01). VTI cardiac index is similar because mean HR is increased in HFpEF patients.

### 3.5. Longitudinal Strain

**Figure 4:**
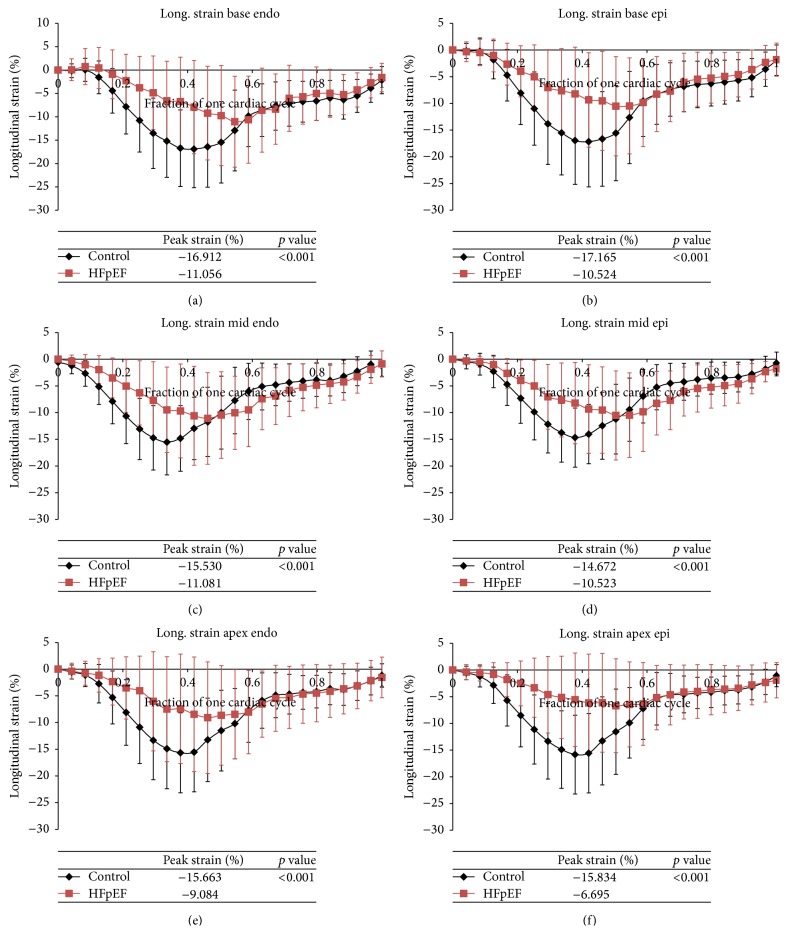
(a)–(f) Average longitudinal strain over one cardiac cycle for control patients (black) versus HFpEF patients (red). Strains at each of the regions of the heart both endocardium (endo) and epicardium (epi) are displayed: base endo, epi (a, b), mid endo, epi (c, d), and apex endo, epi (e, f).

Longitudinal strain is consistently greater in the control patients in all areas of the heart including both the endocardium and epicardium (*p* < 0.001). There is a 4.5% to 9.1% increase in longitudinal strain for the control patients when compared to the diastolic dysfunction patients by region (Table 3). Peak longitudinal strain is also consistently reached later in systole in patients with diastolic dysfunction (Figure 4).

### 3.6. Circumferential Strain

**Figure 5:**
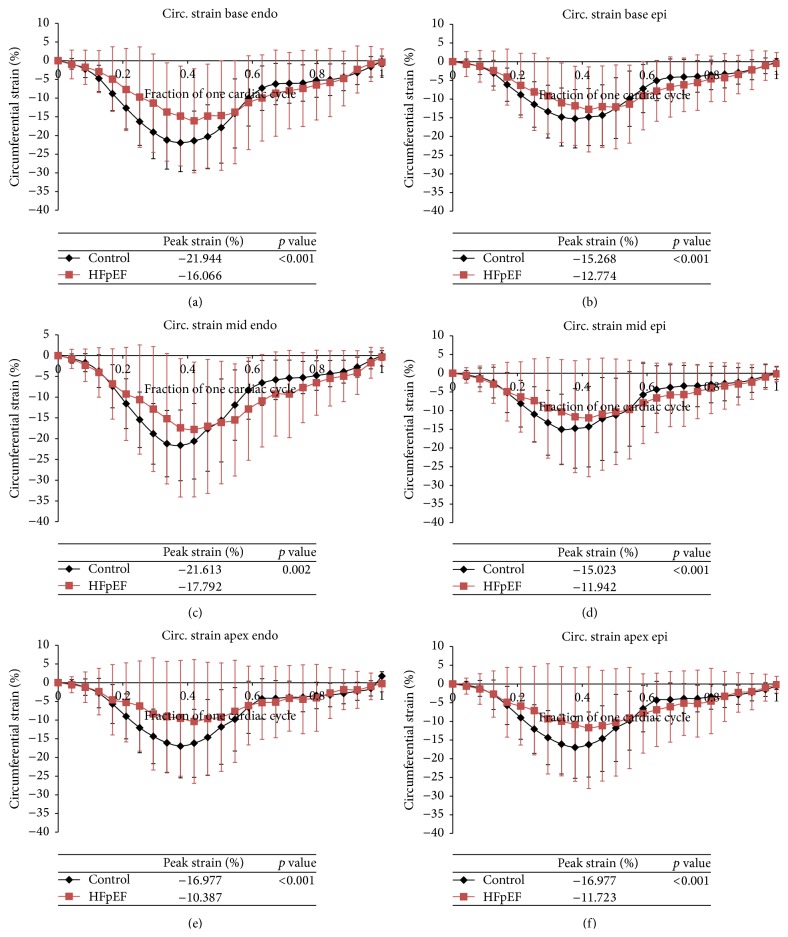
(a)–(f) Average circumferential strain over one cardiac cycle for control (black) versus HFpEF patients (red). Strains at each of the regions of the heart both endocardium (endo) and epicardium (epi) are displayed: base endo, epi (a, b), mid endo, epi (c, d), and apex endo, epi (e, f).

Circumferential strain is consistently greater in the control patients in all areas of the heart including both the endocardium and epicardium (*p* < 0.001). There is a 2.5% to 6.6% increase in circumferential strain for the control patients when compared to the HFpEF patients by region (Table 3). Peak circumferential strain is also consistently reached later in systole in patients with HFpEF (Figure 5).

### 3.7. Rotation

**Figure 6:**
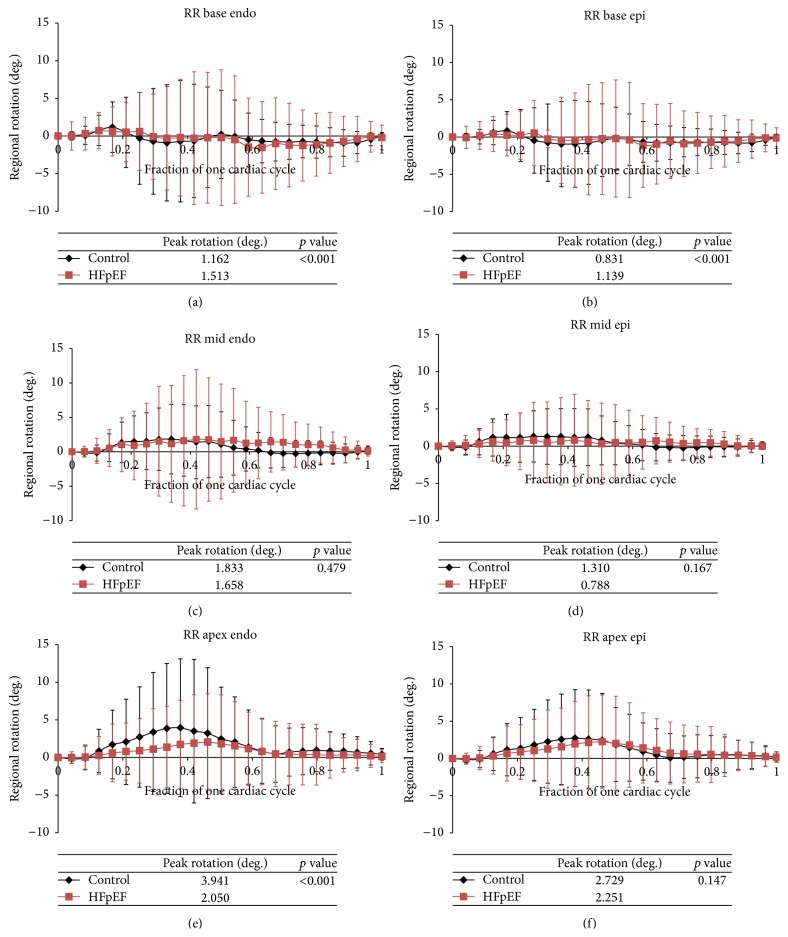
(a)–(f) Average rotation over one cardiac cycle for control (black) versus HFpEF patients (red). Rotation at each of the regions of the heart both endocardium (endo) and epicardium (epi) is displayed: base endo, epi (a, b), mid endo, epi (c, d), and apex endo, epi (e, f).

Rotation of the LV is statistically greater in HFpEF patients at the base of the heart. Rotation is also statistically greater in control patients at the apex of the heart in the endocardium (Table 4). In contrast to the time course of longitudinal and circumferential strain in systole, the time to peak rotation did not differ in controls versus HFpEF patients at the base and mid segments but had a trend towards delay in the HFpEF patients towards the apex (Figure 6).

### 3.8. Strain Rate in Early Diastole

The average longitudinal SRed for controls was 35.5 ± 1.6%/cardiac cycle and for HFpEF it was 18.5 ± 8.5%/cardiac cycle (*p* < 0.001). The average circumferential SRed for controls was 38.8 ± 13.2%/cardiac cycle and for HFpEF it was 18.8 ± 3.9%/cardiac cycle (*p* = 0.005).

### 3.9. Time to Reach Peak Systolic Strain

The conversion factor that converts a percentage systolic interval at a HR of 62 (controls) to a HR of 78 (HFpEF) is 1.13. Therefore each time to peak systole for the control patients was normalized by this factor.

Time to peak longitudinal strain was uniformly delayed in HFpEF and the time to peak rotation was delayed at most locations, but time to peak circumferential strain was in general not prolonged (Table 5).

## 4. Discussion

This study demonstrates that the magnitude of longitudinal, circumferential, and rotational strains during systole is reduced in HFpEF when compared with controls. The time to reach peak systole is reduced in the longitudinal direction and in rotation, but not in the circumferential direction. Early diastolic longitudinal and circumferential strain rate is reduced in HFpEF. Large variability in regional strain among both patients and controls is evident, although the consistency of the findings from base to apex indicates a substantial reduction in both the magnitude of peak longitudinal and circumferential strain and a delay in achieving peak systolic strain in HFpEF. The delay in achieving peak systolic strain and the difference in magnitude of the peak strains are greater in the longitudinal strain components than in the circumferential or rotational strains.

Previous studies demonstrated similar reduction in peak circumferential and longitudinal strains [17, 18]. In a study of HFpEF patients versus asymptomatic patients with diastolic dysfunction Morris et al. [17] demonstrated similar magnitudes of peak strains as our study (longitudinal −0.14 ± 0.03 versus −0.18 ± 0.03 and circumferential −0.15 ± 0.04 versus −0.19 ± 0.04 in HFpEF versus asymptomatic patients), and lower early diastolic strain rates in HFpEF versus asymptomatic patients. No previous study has examined the time-dependence of strain over the normalized cardiac cycle. Our data are the first to suggest both decreased peak and delayed time to onset of systolic contraction.

As found in other studies, rotational strains are less affected than longitudinal and circumferential strains in HFpEF patients. Rotational strains reported here are of much lower magnitude than previously published because the apical region considered here is nearer to the midventricular level than the true apex and because data for LV free wall and septal regions were lumped together. The rotation of the septum is known to be less than the LV free wall. Nevertheless, this study did not show substantial differences in absolute magnitude of rotation between HFpEF and controls but did suggest differences in time to reach peak rotation.

We utilized a small population of HFpEF patients (ten) to allow detailed speckle tracking strain analysis compared with a true control group (eleven patients with normal LV function and no cardiac disease). The HFpEF group is a similarly relatively homogeneous group of heart failure patients reflecting a typical population seen in clinical practice. They have all of the features typically identified with HFpEF patients: they have a high incidence of hypertension, being overweight, half of the patients have diabetes, and more women are included than a typical population of HFrEF patients. All HFpEF patients have elevated diastolic filling pressures with estimated left atrial pressure (18 versus 10) and mean PA pressure (28 versus 13) more than double the controls. The group was selected so that all patients would have Grade II or more diastolic dysfunction by conventional echo criteria. By clinical criteria the patients have heart failure symptoms and all are in NYHA Class II or III and mean BNP level is substantially elevated.

Strain values at each time period show substantial variability marked by the standard deviations of the measurements. This variability arises from variability that undoubtedly occurs from subject to subject in strains based on their age and sex [19], from the noisy speckle tracking data, from regional differences in strains among patients and controls, and from the nature of strain analysis itself because strain is the derivative of LV displacement measurements. Nevertheless, a consistent and highly statistically significant difference in the magnitude, time to peak strain, and pattern of strains (longitudinal versus circumferential, e.g.) was evident in the data. If this pattern of strain analysis is confirmed in a larger data set and with newer and more accurate speckle tracking methods (3D strain analysis) then they may represent a fingerprint for subsequent investigators.

The relationship between changes in passive and active biophysical properties of the myocardium and the resulting LV displacement and strain fields is complex, because the deformation of the LV is strongly dependent on its dimensions and geometry, and on the preload and afterload to which it is subjected. Mathematical models for LV mechanics provide a potentially useful tool for interpreting observations of LV deformation in terms of myocardial biophysics. We previously developed a simple dynamic model of the LV and examined the effects of muscle parameters and circulatory system variables (muscle stiffness, force generation parameters, and peripheral resistance among others) on the time course and magnitude of strain generation [20]. For simulated HFrEF patients, a typical pattern of reduced force generation and increased peripheral resistance generated significant alterations in the magnitude, time course, and pattern of circumferential, longitudinal, and rotational strains. Similar modeling approaches could be used to determine what patterns of diastolic stiffness, systolic force generation, active relaxation properties, peripheral resistance, and parameters affecting the RV and pulmonary circulation generate the distinctive strain characteristics observed in HFpEF patients in this study. Such results could give clues to the pathophysiology of HFpEF and might suggest further experimental studies. For example, an increase in fiber stiffness combined with increased arterial stiffness might be sufficient to generate the observed strain patterns. This would be consistent with the hypothesis that a change in N2B Titin isoform from the N2BA isoform, therefore increasing myofiber stiffness, occurs in HFpEF patients. The delayed early diastolic strain rate might be caused by the reduced compliance of the myocardium so that less elastic energy can be stored, leading to less early elastic recoil and diastolic suction [21]. The difference in time to peak strain between longitudinal and circumferential directions may have a biophysical basis in differences in fiber versus cross-fiber stiffness. Clearly, alternative hypotheses (changes in active relaxation coupled with reduced force generation and increased peripheral resistance, e.g.) might also fit the strain data. These examples suggest the potential for analysis of increasingly accurate and detailed strain data using mathematical models of LV mechanics to probe the mechanisms of HFpEF. Eventually, such models might also be used to predict the effectiveness of therapeutic interventions for HFpEF.

Previous studies have demonstrated a reduction in systolic strains in HFpEF patients [17]. The paradox of reduced systolic strains and preserved EF was explained two decades ago [22]: HFpEF (hypertensive patients in their study) have increased wall thickness so that similar changes in endocardial strains result in greater changes in volume strain. Correspondingly, lower global strain and fiber shortening can generate the same EF in subjects with hypertrophy compared with those who do not have hypertrophy. This was demonstrated in our study where the HFpEF patients had similar EF and similar cardiac index. EF was statistically lower in HFpEF (56 ± 6 versus 50 ± 6%) although still within the normal range. Our data also demonstrate that these patients had increased heart rate and reduced stroke volume. The systolic and diastolic blood pressure were similar indicating that peripheral resistance or arterial elastance was not substantially different. Therefore, the strain data measured here are consistent with the hypotheses that systolic force generation is reduced or alternatively that increased diastolic stiffness alone accounts for reduction in force generation by its effects on the Frank-Starling mechanism and on systolic stiffness. Further analysis by detailed measurements of cardiac motion coupled with a mathematical model may be able to discriminate between these two competing hypotheses.

## Figures and Tables

**Table 1:** Patient characteristics of the control and HFpEF patients.

Patient characteristics	Control (*n* = 11)	HFpEF patients (*n* = 10)	*p* value
Age (years)	39 ± 14	59 ± 12	0.003
Gender (male/female)	2/9	5/5	
Race (Caucasian/African American/other)	11/0/0	10/0/0	
HR (bpm)	62 ± 8	78 ± 13	0.004
Systolic BP (mmHg)	116 ± 23	124 ± 16	0.398
Diastolic BP (mmHg)	69 ± 12	72 ± 13	0.619
BMI (kg/m^2^)	26 ± 4	29 ± 6	0.187
Obesity (BMI > 30) (*n*)	2 (18%)	3 (30%)	
NYHA Classes II-III (*n*)	0 (0%)	10 (100%)	
Diabetes (*n*)	0 (0%)	6 (60%)	
Arterial hypertension (*n*)	1 (9%)	6 (60%)	
Smoking (*n*)	0 (0%)	1 (10%)	
Former smoker (*n*)	3 (27%)	4 (40%)	
Hyperlipidemia (*n*)	1 (9%)	6 (60%)	
NT-pro-BNP (pg/mL) (*n* = 9)	n/a	797 ± 955^*∗*^	
History of coronary artery disease (*n*)	0 (0%)	9 (90%)	
Current beta blocker therapy (*n*)	1 (10%)	10 (100%)	
Current diuretic therapy (*n*)	0 (0%)	10 (100%)	

^*∗*^Normal range 0–100 pg/mL.

**Table 2:** Echocardiographic analysis of control patients versus HFpEF patients.

Hemodynamic parameters	Control (*n* = 11)	HFpEF patients (*n* = 10)	*p* value
MV close time (msec)	471 ± 29	422 ± 61	0.03
IVRT (msec)	102 ± 14	77 ± 15	<0.001
AV open time (msec)	306 ± 33	277 ± 34	0.062
AV close time (msec)	734 ± 157	524 ± 109	0.002
MV open time (msec)	578 ± 167	387 ± 93	0.004
R-R interval (msec)	1068 ± 169	806 ± 121	<0.001
TR jet (mmHg)	18 ± 6	23 ± 13	0.249
IVC pressure (6 beats on IVC with a sniff) (mmHg)	5 ± 0	6 ± 3	0.306
*E*	76 ± 9	94 ± 21	0.019
*E*′	12 ± 3	7 ± 1	<0.001
Left atrial pressure (mmHg)	10 ± 2	18 ± 5	<0.001
Mean pressure in pulmonary artery (mmHg)	13 ± 6	28 ± 9	<0.001
VTI & cardiac index	2.1 ± 0.34	2.2 ± 0.66	0.775
LVOT dia	2.0 ± 0.17	2.0 ± 0.17	0.382
LVOT VTI	19.4 ± 2.1	17.6 ± 3.2	0.12
BSA (m^2^)	1.85 ± 0.23	1.92 ± 0.38	0.61
LV mass (g)	126 ± 31	142 ± 36	<0.01
Stroke volume (mL)	49 ± 11	31 ± 14	<0.01
Ejection fraction (%)	56 ± 6	50 ± 6	<0.01
End diastolic wall thickness (cm)	0.98 ± 0.1	1.18 ± 0.13	<0.01

**Table 3:** Average peak longitudinal and circumferential strain in the base, mid, and apical regions of the heart for both the endocardium (endo) and epicardium (epi) for control versus HFpEF patients.

Myocardium layer, myocardium level	Control peak strain (%)	HFpEF peak strain (%)	Absolute difference between control and HFpEF (%)	*p* value
*Longitudinal strain*				
Base, endo	−16.91 ± 8.25	−11.06 ± 9.72	5.86	<0.001
Base, epi	−17.17 ± 8.46	−10.52 ± 9.29	6.64	<0.001
Mid, endo	−15.53 ± 6.13	−11.08 ± 8.58	4.45	<0.001
Mid, epi	−14.67 ± 5.54	−10.52 ± 7.52	4.15	<0.001
Apex, endo	−15.66 ± 7.49	−9.08 ± 10.44	6.58	<0.001
Apex epi	−15.83 ± 7.38	−6.70 ± 8.80	9.14	<0.001
*Circumferential strain *				
Base, endo	−21.94 ± 7.73	−16.07 ± 13.93	5.88	<0.001
Base, epi	−15.27 ± 7.82	−12.77 ± 11.38	2.49	<0.001
Mid, endo	−21.61 ± 8.49	−17.79 ± 16.23	3.82	0.002
Mid, epi	−15.02 ± 7.97	−11.94 ± 15.74	3.08	<0.001
Apex, endo	−16.98 ± 10.64	−10.39 ± 16.53	6.59	<0.001
Apex epi	−16.98 ± 10.64	−11.72 ± 16.25	5.25	<0.002

**Table 4:** Average peak rotation in the base, mid, and apical regions of the heart for both the endocardium (endo) and epicardium (epi) for control patients versus HFpEF patients.

Myocardium layer, myocardium level	Control peak rotation (degrees)	HFpEF peak rotation (degrees)	Absolute difference between control and HFpEF (degrees)	*p* value
Base, endo	1.16 ± 3.36	1.51 ± 6.07	0.35	<0.001
Base, epi	0.83 ± 2.58	1.39 ± 5.62	0.56	<0.001
Mid, endo	1.83 ± 5.06	1.66 ± 7.54	0.18	0.479
Mid, epi	1.31 ± 3.44	0.79 ± 5.12	0.52	0.167
Apex, endo	3.94 ± 9.16	2.05 ± 6.40	1.89	<0.001
Apex, epi	2.73 ± 6.50	2.25 ± 6.25	0.48	0.147

**Table 5:** Time to reach peak systole (entire cardiac cycle length 1.0 as in Figure 6, e.g.). At each location, the normalized control was compared with HFpEF.

	Base		Mid		Apex	
	% cardiac cycle ± std. dev.	*p* value	% cardiac cycle ± std. dev.	*p* value	% cardiac cycle ± std. dev.	*p* value
Longitud. strain						
Endo						
Normalized control	0.450 ± 0.014	<0.001	0.404 ± 0.013	<0.001	0.404 ± 0.013	<0.001
HFpEF	0.525 ± 0.012		0.442 ± 0.013		0.442 ± 0.013	
Epi						
Normalized control	0.451 ± 0.014	<0.001	0.403 ± 0.013	<0.001	0.403 ± 0.013	<0.001
HFpEF	0.485 ± 0.012		0.485 ± 0.012		0.495 ± 0.012	

Circumfer. strain						
Endo						
Normalized control	0.404 ± 0.013	0.604	0.403 ± 0.014	0.739	0.404 ± 0.014	0.507
HFpEF	0.401 ± 0.013		0.401 ± 0.013		0.400 ± 0.013	
Epi						
Normalized control	0.404 ± 0.013	0.604	0.350 ± 0.010	<0.001	0.404 ± 0.014	0.507
HFpEF	0.401 ± 0.013		0.401 ± 0.013		0.400 ± 0.013	

Rotation						
Endo						
Normalized control	0.399 ± 0.107	0.001	0.439 ± 0.097	0.260	0.390 ± 0.084	0.007
HFpEF	0.591 ± 0.125		0.493 ± 0.116		0.565 ± 0.171	
Epi						
Normalized control	0.436 ± 0.079	0.014	0.443 ± 0.101	0.483	0.408 ± 0.096	0.017
HFpEF	0.556 ± 0.122		0.473 ± 0.090		0.564 ± 0.171	

## Limitations

A single observer performed all of the strain computations and interobserver variability was not determined. Previous studies have estimated the interobserver variability to be small in comparison to other sources of variability in the strain data [17]. Furthermore, use of one observer results in more consistency in technique throughout the speckle tracking process. Interobserver variability should be similar in HFpEF and controls. As a small single institution study, this work does not include the number of patients or heterogeneity of HFpEF population accrued in other studies [17, 18]. Measurements of LV deformation do not directly indicate the mechanical and contractile properties of the LV, since deformation is sensitive to afterload and preload conditions. In future work, mathematical models of LV mechanics will be combined with observations of LV strain to overcome this limitation.

In summary, HFpEF patients have delayed onset and reduced magnitude of systolic strain generation coupled with reduced early diastolic strain rate. These data are consistent with the hypothesis that an increase in passive diastolic stiffness of the myofibers (change in Titin N2B isoform) leads to both reduced systolic contraction and less elastic recoil during early diastole. Subsequent modeling, strain analysis, and cellular and molecular studies should examine this hypothesis further. Future improvements in speckle tracking techniques, for example, 3D tracking, have the potential to lead to better understanding of the pathophysiology of HFpEF and to better methods for monitoring the effects of therapy.
